# Apparent Trifles: Their Importance in Dental Practice

**Published:** 1904-04

**Authors:** F. Milton Smith

**Affiliations:** New York


### APPARENT TRIFLES: THEIR IMPORTANCE IN DENTAL PRACTICE.[1]

BY DR. F. MILTON SMITH, NEW YORK.

"He that is faithful in that which is least is faithful also in much." More than nineteen centuries have passed since these words came from the lips of the Great Teacher, and from that time to the present, in forms almost without number, the truth has been restated.

In no field is this teaching more clearly demonstrated than in our work. Were it not for the fact that many of our "craft" who have passed to "that bourne whence no traveller returns" realized the force of this truth, we to-day would not meet with the measure of success we do.

If I speak to-night to any young man who is not willing to give the utmost attention to any detail that may in any way

---

[1] Read before the American Academy of Dental Science, Boston, Mass., March 2, 1904.

effect the accomplishing of the work he may have in hand, I would urge him not to waste his time trying to build up a dental practice, but immediately get a position as trolley-car motorman or conductor. While there is plenty of room for young men who are determined to succeed, and are willing to pay "the price thereof," there is, indeed, only just standing-room for the young man who considers details beneath his notice.

How familiar to our thought is the story of the battle lost through the want of a horseshoe-nail, yet how many there are who are willing to pay almost any price to succeed, but will pass by that great stepping-stone to success,—namely, attention to details, with scarcely a thought.

One has but to read the average article in the dental journal to be persuaded that this lack of attention to detail is not confined to the mere boys in the profession.

In a recent issue of a dental journal there were two articles by good men. After very careful reading both were thrown aside as worthless, because in each article an important detail was missing.

Apparent trifles are exceedingly important in their bearing upon the comfort or discomfort of the patient; and to the lack of appreciation of their importance may be charged the loss of the good opinions of many patients.

All other things being equal, any genteel patient will select the dentist whose office and surroundings are pleasing.

While a man of skill and integrity will sometimes gather around him a good practice, even if his office and reception-room are not such as might be expected, he would nevertheless attract more people if the surroundings were pleasant.

When a patient enters the office of one practitioner he is pleasantly impressed. There is a place for everything and everything is in its place. In another everything is confusion. The one has a flower or two on the mantel. The other has everything else in creation but a flower or two. In the one case the operator is strictly neat and clean in his personal appearance. There is about him neither the odor of pipe, cigarette, beer, nor onions. He cares for his own mouth and teeth and employs a good dentist: therefore his breath is not loaded with the odors of pyorrhœa or abscessed teeth. In fact, he studies to keep himself absolutely free from odors, with the possible exception of a faint suggestion of cashmere bouquet soap with which he washes his hands very often. In the other the reverse is true.

In the one case the operator opens a drawer of his cabinet, whence he takes a towel or napkin which is immaculately clean. If the other opens a drawer there is revealed in its recesses two or three extracted teeth, some broken pieces of wax with which the floss is waxed, a broken hand-piece, some old scratched mirrors, and a miscellaneous assortment of other things too numerous to mention.

In the latter case the operator may have a machine on his table conspicuously marked "Sterilizer," and make a pretence at antiseptic work, but the aforesaid drawer is silent yet forcible evidence to the contrary.

It was my privilege on one occasion to visit a friend who made quite a pretence at things aseptic. In order to demonstrate to me his methods, he opened his aseptic napkin-box, then moistening his finger with his tongue, after the fashion of a trolley-car conductor when separating slips, picked up one of the napkins.

He then demonstrated to me his method of rolling absorbent paper points for root drying, pursuing the same course as to moistening the fingers, so that the paper would roll well. He said after they were rolled they were sterilized. I think that was right; they needed it.

In another case a dentist, who is also a great preacher of antiseptic methods, requested me to wash my hands before examining the mouth of a patient whom he was kind enough to let me see; yet he was so busy superintending my proper sterilization that he forgot to change the napkin on which he was constantly wiping the mouth-mirror, although he changed patients four times while I was present.

As to the apparent trifles which affect the comfort of the patient. The careful man sees to it that the chair is adjusted to the comfort of his patient as well as to his own convenience. A few minutes carefully spent in this seemingly unimportant detail will pay large interest on the investment. The patient will almost invariably thank you to make an application of some cooling emollient to the lips, if they are at all dry or have a tendency to crack.

The rubber dam, if applied before excavating, will save much valuable time, though there may be room for question as to whether the average patient would not prefer to have its application delayed until the last moment. When ready to apply it there is room to demonstrate your ability to do things carefully. It will pay almost always to make six holes through the rubber and surround that number of teeth.

I think the average dentist should have the dam applied to his own teeth by some careless man at least once a week. He would then use care in applying it for others. Ordinarily there should be no pain and very little discomfort through its application. In the cold weather if you will place the clamp in the forceps and then hold the clamp over your Bunsen burner just long enough to warm it, your patient will bless you. If, in carrying your ligature between the teeth, you are careful not to let it strike the gum suddenly, your patient will appreciate it. If you are about to fill an ordinary cavity, it is absolutely cruel to ligate several teeth, carrying the ligature up above the natural gum line.

When preparing a cavity it is not necessary to have it so dry that the tears will course down the patient's cheeks because of the pain caused by the dryness and low temperature of the room, as compared with the body. A pledget of cotton dipped in carbolic acid and passed through the lamp flame, then to the cavity, will change the expression on the face as if my magic. If applied cold the patient will dread it as much as the pain you seek to control. If, when applying the dam clamp, you fold two little pieces of muslin and lay them between the clamp and cheek, your patient will appreciate it.

If you are filling a large sensitive cavity with amalgam, it will lessen the discomfort greatly if you will warm your plugger or burnisher each time you use either of them. If it is a gold filling you have made, you will find much less complaint from your patient during the finishing if your bur is run into a bit of beeswax and your disk against a piece of soap or coated with vaseline before use.

A corundum point is much less annoying if coated with vaseline instead of water, and answers as well when used on metal.

When you are ready to remove the dam your patient will leave you in a happier frame of mind if you do not jam the clamp down unnecessarily on the already sore gum, nor remove it so suddenly that every particle of blood shall seem all at once to be trying to get to the gingival margin. Having removed the clamp and dam in the most approved method, your patient will be exceedingly grateful to you if you will thoroughly spray the gums with an antiseptic, or syringe carefully with warm water and antiseptic. In order to have this water always at the right temperature you need a small Bunsen gas-burner with a rack to hold a glass of water over it, placing between the flame and the glass a piece of asbestos mat. If you do not have something of this kind, one of two things will occur,—either you will torture your patient by throwing cold water into a sensitive tooth or else you will waste an enormous amount of time between the warm water faucet and your chair. As to the various methods of relieving the sensitivity, each operator will have his pet method. Your essayist finds that the cases differ materially as to their responsiveness to drugs. Besides the many in common use, I find the plan recently suggested by Dr. L. C. Bryan, of Basel, Switzerland, to be very useful in many cases.

In a communication through The New York Institute of Stomatology he suggests a pledget of cotton saturated with carbolic acid, which, after being placed in the cavity, is touched with a metal point which is heated sufficiently so that when applied to the moist cotton a vapor is produced. This repeated several times works beautifully in many cases.

Lack of time forbids my recounting more of the apparent trifles, and they are almost without number, which affect the comfort of the patient. I will therefore call attention to some which have to do with the quality of the service we render our patients.

Why is it that the magnificent gold filling in the distal face of a certain bicuspid tooth has failed?

The one in the mesial face of the adjoining tooth still is doing service, although five years longer in place. The former filling bears evidence of having been made by a clever mechanic, yet has not saved the tooth. The reason for failure is that the operator considered that a blow from an automatic mallet upon a piece of cohesive gold, driven directly against the cervical wall, was a trifle not to be reckoned on. His neighbor who placed the other filling in position considered it wise to see to it that, previous to any blow being struck by a mallet, he had first placed a large mass of non-cohesive gold against the cervical wall. He may have also thought that suggestion a valuable one (first made public, I think, by Dr. S. G. Perry, of New York City) which consists in placing a very large piece of non-cohesive gold in the cavity, following with a large ball of bibulous paper, which paper is packed with a large pointed plugger, carrying before it the soft gold. The paper then being removed, the gold is found safely carried to the floor of the cavity without being punctured or cut through with the plugger point. It is then with smaller points very carefully condensed without the mallet. After making two or three layers thus, the cohesive gold may be malleted *ad libitum.*

Many a young man has wondered why his filling did not finish up like the filling of some excellent operator whose work he has admired. If you see him tell him that it is not that his ideal operator is so much more skilful than he. It is because he got tired out too soon and failed to appreciate that all that was needed to make his filling finish perfectly was two or three large pieces of gold, covering the whole surface of the filling like a blanket carefully laid on with a broad, flat, almost smooth-face plugger; this to be followed by a very small point with which the surface is condensed.

Tell him also, for his encouragement, that his ideal operator has not skill enough to put a perfect filling in the cavity where he failed yesterday, with no more room than he had. It is not that his ideal is so much more expert as a mechanic. It is that he knows that to fill the cavity perfectly he must have room, and without it he will not attempt the task.

Who has not seen the disreputable-looking gold-band porcelain crown, showing more gold than porcelain? It is the simple result of lack of attention to details (apparent trifles). First, the gum was not crowded up on the labial side when the root was ground away, therefore it could not be ground above the natural gum line without injuring the gum. Second, the root was not so reduced in diameter at the exposed portion as to make it possible to so fit a band that it would hug the root snugly under the gum. Third, the band was not cut off even with the already too long root. Fourth, a thirty-guage piece of gold was used to cover the root instead of forty. Fifth, the porcelain did not fit perfectly against the cap. Result, a frightful disfigurement. Why has the patient found it less useful than ornamental? Another trifle. Care was not taken that it should be sufficiently wide at the swell portion so that it would crowd closely between the adjoining teeth when set. Had this been done it would have been comfortable.

Some one says, "I get along well with the operative side, but my plate work is not successful. The plate I put in yesterday would not stay up until I sprinkled it with gum tragacanth." Of course it wouldn't. You were too hurried, and concluded to let a compound impression do when it should have been a plaster one. "Yes, but the one I put in the day before did not stay up, and it was made from a plaster impression." The reason for its failure was that you did not make a groove in the impression across the roof of the mouth, so that the extreme back edge of the plate would surely go up to place; or maybe you did not take time to notice that the hard palate is very hard in the centre of that mouth, and the hardness reaches almost to the alveolar ridge in front. A very large piece of thin air-chamber metal should have been carefully placed on the model, covering the hard territory before packing the rubber. It is more than likely you did not wait for the flask to cool thoroughly before opening; therefore there was a slight drawing away from the centre. Again you have treated as apparent trifles the valuable suggestions which have appeared from time to time in the journals. In other words, the majority of your failures are the results of neglecting details.

Not the least among the apparent trifles is the habit of wasting time which prevails among dentists.

If one's fee is six dollars per hour, his minutes are worth just ten cents each. Imagine a poor man sitting on a cracker barrel in a country store throwing a ten-cent piece out of the window every minute, and you have a forcible illustration of the dentist whose fee is six dollars per hour and upward, when he wastes his time. I am not familiar with your habits in this town, but in the one whence I came many dentists have no time to read the dental journals, yet they will squander more time every day in reading the details of the latest social scandal, murder trial, or other trash than would suffice to read any one of the dental journals. Were the average man to be impressed with the fact that half an hour spent each day in this useless reading would in the course of a year run away with eighteen days of eight hours each, he would, I think, put his time to better use. Many of this class would not think they could possibly take a three weeks' vacation.

Since the dentist must, with his own brain and hand, do almost everything that is done in his office, it is of the utmost importance that he shall make every minute count for its full value. He must certainly, as soon as he can possibly afford it, employ some one to do the little things about the office. It is the habit of some excellent practitioners to take time for which they are charging their patients ten or twelve dollars an hour, and use that time in folding, rolling, and cutting gold, rolling up paper pellets, and such like work, when a girl for five dollars a week can do it as well.

A little thing worth attention is the time lost between patients. It is absolutely impossible to tell just how long a given operation will take; therefore it is best to make appointments for one hour, one and a half, or two hours each, always saying to the patient that the next sitting will be for one of these stated periods. If the appointment is made over the telephone or by mail, a most excellent way is to mail to the patient a duplicate slip, each part of which contains the date, hour, and time reserved. One is retained by the patient, and the other, which contains the words, "Mrs. —— accepts the appointment," is returned by mail. This plan obviates the embarrassment of having a vacant chair for an hour unexpectedly. It also impresses the patient with the value of our time.

For suggestions in this direction I am greatly indebted to my present associate and friend, Dr. George S. Allan.

The appointment having been made for, say, one and one-half hours, it is well at once to proceed with the preparation of a cavity or other piece of work that can surely be done in that time. This will usually leave a few minutes before the next patient. These minutes can almost invariably be used to benefit to the patient in removing calculus or in polishing the teeth. At the next sitting the same course is pursued, so that by the time we have the fillings all made we have the teeth cleaned and polished or so nearly finished that we can judge very closely as to how much time will be required for its accomplishment.

Had we spent two hours at the first sitting in cleansing and polishing, we could not conscientiously have made all our appointments come out evenly.

By this method we have practically saved the time required for this work, which is no small item.

Another waste of time is caused by the practice of permitting lady patients to remove their hats and wraps in the operating-room. This needs no further comment other than to say that ten minutes lost at each change of patients means, if repeated six times in a day, just one hour out of your working day.

Lest I weary you, I will thus abruptly close, leaving you to think out the many, many apparent trifles which hitherto you have overlooked, hoping that you, as well as I, may have indelibly stamped upon our minds and hearts the value of time as conceived by one who says,—

> "Across the years I fly on tireless wing;
> No rest I ever know; on, on I go,
> Nor stay my ordered flight for friend or foe.
> Once past I never shall return to bring
> Again my gifts so often spurned, for, lo,
> Each moment is a bridge that's burned.
> I know no haste, yet in my flight outspeed the light,
> While out of seconds ageless cycles grow.
> Who knows my name and freely will
> Bestow on me his best of hand, and heart and mind,
> I'll give him true success and clearly show
> The secret of my power to bless mankind;
> He me enjoys who me employs aright.
> My name is Now. Lay hold with all thy might."

